# Triathletes and Urinary Incontinence: An Investigation of Prevalence and Associated Factors

**DOI:** 10.1002/nau.70145

**Published:** 2025-09-08

**Authors:** Tais Schwamberger, Thuane Huyer da Roza, Eliane Regina Mendoza Arbieto, Isabela Cardoso Ferreira, Letícia Beatrice Tramontin Schuler, Luiz Henrique Cabral Duarte, Soraia Cristina Tonon da Luz

**Affiliations:** ^1^ Laboratório de Biomecânica, Centro de Ciências da Saúde e do Esporte (CEFID) Universidade do Estado de Santa Catarina (UDESC) Florianópolis Brazil

**Keywords:** athletes, exercise, logistic models, pelvic floor, stress urinary incontinence

## Abstract

**Aims:**

This study aimed to investigate the prevalence of urinary incontinence (UI) among Brazilian female triathletes and to identify associated factors, focusing on demographic, obstetric, and sports‐related variables.

**Methods:**

A cross‐sectional study was conducted with 90 female triathletes. Data on age, body mass index (BMI), pregnancy history, parity, delivery type, training frequency, and weekly training volume were collected through in‐person interviews and an online questionnaire. UI severity was assessed using the International Consultation on Incontinence Questionnaire – Short Form (ICIQ‐SF). Ordinal logistic regression was used to analyze factors associated with UI severity, and multinomial logistic regression examined associations between training volume and UI type.

**Results:**

The prevalence of UI was 43.3%. Stress urinary incontinence (SUI) was the most common type (25.6%). A greater number of deliveries was associated with increased UI severity (OR = 1.577; 95% CI: 1.047–2.374), while higher training frequency was protective against UI (OR = 0.761; 95% CI: 0.607–0.954). Increased running volume was associated with greater odds of presenting mixed UI (OR = 1.004; 95% CI: 1.001–1.006). Weekly training frequency was inversely associated with both stress and mixed UI.

**Conclusions:**

UI was reported by 43.3% of triathletes, with SUI being the most prevalent type at 25.6%. Parity was the only sociodemographic factor significantly associated with UI. Higher weekly frequency in all disciplines appeared to reduce the likelihood of UI, especially SUI. In contrast, running volume showed a minimal or no association with an increased risk of MUI.

## 1 Introduction

Over the past decade, studies have shown a high prevalence of urinary incontinence (UI), especially stress UI (SUI), among young and nulliparous women, particularly those involved in sports activities [1, 2, 3, 4]. The prevalence of UI varies based on the type of sport practiced. Athletes participating in high‐impact sports, such as trampoline and gymnastics, exhibit a high prevalence rate that can reach up to 80%. In contrast, the prevalence is considerably lower, at around 10.9%, among those involved in low‐impact sports, such as cycling [5, 6]. Additionally, training intensity and volume also influence the risk of SUI, with athletes who undergo higher training loads being more predisposed to this condition [7, 8].

During exercise, bladder pressure may exceed the urethral closure pressure, resulting in urine loss [9]. However, the exact physiopathology of SUI in athletes remains unclear. Bø [10] suggested that repetitive loading of the pelvic floor can lead to muscle fatigue and/or tissue strain, which contributes to urine leakage during repetitive high‐impact activities, such as running [11]. In fact, some researchers have reported that SUI tends to occur mainly during the middle or at the end of training sessions [12]. Beyond the physiological mechanisms, SUI affects the sports confidence and quality of life of athletes [13, 14], leading some women to quit sports [5]. Despite this, many athletes consider SUI a natural consequence of the sport and rarely seek help.

There are a few studies investigating the prevalence of UI in female triathletes. Our search identified only the study by Yi et al. [15], which assessed 311 American female triathletes, aged between 35 and 44 years, and found a prevalence of 37.4% for SUI, 16.1% for urgency UI, 28% for anal incontinence (AI), and 5% for pelvic organ prolapse (POP) [15]. Triathlons have a unique characteristic in that they combine three sports: running, which can be classified as high‐impact, cycling, and swimming, which are considered low‐impact. The multifactorial nature of the sport and the various mechanical loads involved during the activity underscore the need to investigate the impact of the sport on the function of the PF muscles in female triathletes.

To date, there are no studies on the prevalence and associated factors of UI in female triathletes in the Brazilian population. Therefore, the main objective of this study was to investigate the prevalence and associated factors of UI in Brazilian women who practice triathlon, to contribute to the development of strategies for preventing UI in this population.

## 2 Materials and Methods

### 2.1 Study Design and Population

This study is a cross‐sectional observational study conducted between April and November 2024. Women aged 18 to 50 years, with regular triathlon practice for at least 1 year before the study, were screened and included. Data collection was carried out during triathlon competitions in the city of Florianópolis, training sessions of triathlon groups, and through online dissemination (Google Forms via email and Instagram). Women with recent surgery impacting training, pregnancy or abortion in the past year, neuromusculoskeletal diseases, active urinary tract infection (self‐reported burning during urination), or atrophic vaginitis (self‐reported) were excluded. The study was approved by the Human Research Ethics Committee of the State University of Santa Catarina (approval number 6.547.406; CAAE 74004823.0.0000.0118) and conducted in accordance with the ethical principles outlined in the Declaration of Helsinki.

### 2.2 Data Collection

All participants completed an evaluation form designed to collect information about the athlete's profile, considering aspects such as general health, triathlon practice, and menstrual cycle. The form requested sociodemographic data, including date of birth, occupation, education level, weight, height, marital status, and information on the eligibility criteria. Data regarding triathlon experiences were also collected, including practice duration, frequency, and the length of training sessions in swimming, cycling, and running, as well as details about participation in annual competitions.

### 2.3 Assessment of Urinary Incontinence and Outcomes

The primary outcome was UI, assessed using the International Consultation on Incontinence Questionnaire—Short Form (ICIQ‐SF) [17]. The ICIQ‐SF is a simple, reliable, and validated questionnaire that is widely used in clinical practice and research. The questionnaire consists of four main questions. The first addresses the frequency of urine loss, investigating how often the participant experiences UI episodes in the last 4 weeks. The second question evaluates the perception of the amount of urine lost during these episodes. The third question evaluates the impact of UI on quality of life, considering how much it interferes with everyday life.

Additionally, there is a fourth self‐diagnostic item that asks, “When does urine leak?”, allowing participants to identify specific situations associated with urine loss. Responses to this question were used to classify the UI as stress UI (SUI), urgency UI (UUI), or mixed UI (MUI), based on the participants' responses. Finally, the total score indicating the severity of UI was calculated by summing the first three questions, resulting in a score ranging from 0 to 21. A higher score indicates greater severity of UI. The severity scores were categorised as mild (1–5), moderate (6–12), severe (13–18), and very severe (19–21) [17].

The secondary outcomes were to verify the influence of training volumes and training frequency on the prevalence of different types of UI. The variable “training volume” was analyzed according to Rodríguez‐López et al. [16], measuring it in minutes of training per week. Thus, this study analyzed the training volume (minutes per week) and training frequency (number of training days per week) within each triathlon discipline.

### 2.4 Statistical Analysis

For data analysis, the IBM SPSS software, version 20, was used. Descriptive data analysis was conducted using mean and standard deviation (SD) for numerical variables and percentages for the sociodemographic profile. The association between variables was assessed using Odds Ratio (OR). The prevalence and association between variables such as age, parity, pregnancies, BMI, and training volume were analyzed using ordinal logistic regression, starting with a crude analysis, followed by an adjusted analysis, ensuring proper data organization and interpretation to answer the study's objectives.

The influence of specific and total training volumes and frequencies on the prevalence of different types of UI was tested through simple and multiple multinomial logistic regression. Initially, each predictor variable was tested individually to verify its association with the outcome and define its inclusion in the adjusted model through the Omnibus test for model fit assessment.

### 2.5 Sample Size

The sample size calculation for the analysis was performed based on the recommendation of Peduzzi et al. [18], who suggest that, to ensure the stability of the estimates, the ideal is to have between 10 and 15 individuals per predictor variable in multiple logistic regression models. The sample of 90 participants, in a compromise analysis in the G*Power 3.1.9.7 software for logistic regression and considering a prevalence of 39% of UI in the triathletes evaluated, indicated the probability of alpha and beta error of 0.046, and a power of 0.953. Thus, the study sample proved to be suitable for multiple logistic regression analyses and capable of providing high statistical power.

## 3 Results

Among the 143 athletes approached, ten were excluded due to age (> 50 years), and 43 were excluded for not completing the questionnaires. Thus, the study included 90 women, with a mean age of 38.1 ± 6.7 years and a mean BMI of 22.3 ± 2.2. Of these 90 athletes, 73 were recruited during triathlon competitions, 5 were referred by coaches, and 12 completed the questionnaires online.

In total, 39 (43.3%) triathletes self‐reported as incontinent. Among them, 23 (25.6%) reported SUI, three (3.3%) had UUI, and nine (10%) had MUI. Additionally, four participants (10.4%) did not respond to the question that asked them to identify the type of UI. Among the incontinent triathletes, 25 (27.8%) reported urine loss during sports practice. Regarding UI severity, according to the ICIQ‐SF, 23 (58.9%) participants had mild UI, 14 (35.9%) had moderate UI, and 2 (5.1%) had severe UI.

Table 1 presents the socioeconomic and reproductive/clinical variables of the participants, as well as their training pattern. Data on UI prevalence and childbirth types are also provided. Regarding the training patterns, it is noted that there were considerable variations, with routines ranging from 1 to 10 training sessions per week and durations from 30 min to 8 h per day.

**Table 1:** Descriptive analysis of the sample including age, BMI, pregnancy and parity history, UI presence, menstrual cicle, and characteristics, triathlon practice duration, training frequency, and volume (*N* = 90).

Variable	*n* (%)	Mean ± standard deviation
**Socioeconomic and reproductive/clinical variables**		
Age	—	38.1 ± 6.7
BMI	—	22.3 ± 2.2
Pregnancy	35 (38.8%)	—
Parity	32 (35.5%)	—
UI	39 (43.3%)	—
SUI	23 (25.6%)	—
UUI	3 (3.3%)	—
MUI	9 (10%)	—
Normal delivery	6 (6.7%)	—
Cesarean delivery	29 (32.2%)	—
Do you menstruate (*n* = 87)		
Yes	55 (61.1%)	
No	32 (35.6%)	
Regular menstrual cycle	33 (60%)	
Irregular menstrual cycle	13 (23.6%)	
No answer	9 (16.4%)	
Marital status (*n* = 88)		
Married	41 (46.6%)	
Single	12 (13.6%)	
Stable union	17 (19.3%)	
Divorced	15 (17%)	
Engaged	1 (1.1%)	
Widowed	2 (2.3%)	
Educational level (*n* = 88)		
High school graduate	12 (13.6%)	
University graduate	22 (25%)	
Postgraduate level	54 (61.4%)	
**Training pattern**		
Triathlon practice duration (months)	—	54.5 ± 49.36
Weekly running frequency (Sessions/week)	—	3.5 ± 1.01
Running volume (Minutes/week)	—	371.0 ± 308.96
Weekly cycling frequency (Sessions/week)	—	3.3 ± 0.84
Cycling volume (Minutes/week)	—	486.8 ± 326.19
Weekly swimming frequency (Sessions/week)	—	3.1 ± 1.24
Swimming volume (Minutes/week)	—	299.1 ± 304.37
Weekly training frequency (All sports, Sessions/week)	—	9.9 ± 1.99
Weekly training volume (All sports, Minutes/week)	—	1156.88 ± 708.36

*Note:* BMI (Body Mass Index), SD (Standard Deviation), Weekly frequency (number of days per week the athlete trains), Volume (Minutes per Session * Weekly Frequency), UI (urinary incontinence), SUI (stress urinary incontinence), UUI (urgency urinary incontinence), MUI (mixed urinary incontinence).

The Crude logistic regression analysis (Table 2) showed a significant association between UI and parity, and UI and weekly training frequency. UI and age, as well as UI and BMI showed no statistically significant associations. The adjusted analysis of these variables (multiple ordinal logistic regression) confirmed that parity is an associated risk factor for UI. At the same time, the weekly training frequency of all sports is a protective factor for UI.

**Table 2:** Crude analysis of associated factor variables for urinary incontinence using an ordinal logistic regression model and the adjusted model.

Variable	OR (95% CI)	Omnibus test
**Simple ordinal logistic regression**		
Age	1.009 (0.183–18.158)	*p* = 0.773
BMI	1.003 (0.994–1.011)	*p* = 0.573
Pregnancies	1.443 (0.987–2.109)	*p* = 0.058
Parity	1.619 (1.070–2.449)	*p* = 0.021*
Weekly training frequency (All sports)	0.756 (0.605–0.944)	*p* = 0.010*
Weekly training volume (All sports)	1.000 (0.999–1.000)	*p* = 0.702
Surgical history		*p* = 0.426
Yes	1	
No	2.408 (0.242–23.908)	
		
**Multiple ordinal logistic regression**		
Adjusted model		*p* = 0.003*
Parity	1.577 (1.047–2.374)	
Weekly training frequency (All sports)	0.761 (0.607–0.954)	

Abbreviations: 95% CI, 95% confidence interval; BMI, Body mass index; OR, Odds ratio.

* Statistically significant variable in the crude analysis; Severe UI was used as the reference category.

To determine which sports could be associated with risk factors for the different types of UI (SUI, UUI, or MUI), a multinomial logistic regression analysis was performed. The crude analysis revealed that running volume (minutes/week), weekly swimming frequency, and weekly frequency (all sports) were significantly associated with the different types of incontinence (Table 3).

**Table 3:** Analysis of predictor variables for the outcome type of urinary incontinence using multinomial logistic regression.

**Variable**	**SUI OR (95% CI)**	**UUI OR (95% CI)**	**MUI OR (95% CI)**
**Simple multinomial logistic regression**			
Weekly running frequency (Sessions/week)	0.664 (0.339–1.300)	1.373 (0.724–2.602)	0.701 (0.271–1.811)
Running volume (Minutes/week)	0.997 (0.997–1.002)	1.001 (0.998–1.005)	1.002 (1.000–1.004)*
Weekly cycling frequency (Sessions/week)	0.803 (0.426–1.512)	0.603 (0.133–2.739)	0.500 (0.194–1.290)
Cycling volume (Minutes/week)	1.000 (0.999–1.002)	0.998 (0.992–1.004)	1.001 (0.999–1.003)
Weekly swimming frequency (Sessions/week)	0.504 (0.278–0.914)*	0.727 (0.234–2.253)	0.401 (0.154–1.045)
Swimming volume (Minutes/week)	0.999 (0.998–1.001)	0.999 (0.994–1.004)	0.999 (0.996–1.002)
Weekly training frequency (All sports)	0.694 (0.510–0.946)*	0.994 (0.544–1.819)	0.603 (0.383–0.950)*
Weekly training volume (All sports)	1.000 (0.999–1.001)	1.000 (0.998–1.002)	1.000 (1.000–1.001)

**Multiple multinomial logistic regression**			
**MODEL 1**			
Running volume (Minutes/week)	0.999 (0.997–1.002)	1.001 (0.996–1.006)	1.004 (1.001–1.007)*
Weekly swimming frequency (Sessions/week)	0.581 (0.234–1.443)	0.648 (0.112–3.767)	1.353 (0.343–5.337)
Weekly frequency (All sports)	0.891 (0.534–1.487)	1.110 (0.413–2.982)	0.440 (0.202–0.959)*
**MODEL 2**			
Running volume (Minutes/week)	1.000 (0.998–1.003)	1.002 (0.998–1.006)	1.004 (1.001–1.006)*
Weekly frequency (All sports)	0.695 (0.505–0.956)*	0.909 (0.482–1.715)	0.482 (0.281–0.825)*

*Note:* The category without UI was used as the reference category.

Abbreviations: 95% CI, 95% confidence interval; OR, Odds ratio.

* Statistically significant variable in the crude analysis.

The model with the variables running volume and weekly frequency (all sports) passed the multicollinearity test, and the multinomial logistic regression was performed, as shown in Table 3. In the adjusted analysis, the variable weekly swimming frequency was not statistically significant and was removed from the final model.

The adjusted model 2 revealed that weekly training frequency (all sports) is a protective factor for SUI and MUI. Meanwhile, higher weekly running volume was found to be an associated factor for MUI in female triathletes, although with low relevance.

## 4 Discussion

Of the 90 triathletes evaluated, there was a prevalence of 43.3% of UI, with the most common type being SUI. Additionally, nearly 25% of the triathletes reported urinary leakage during sports activities. In this study, parity was the only sociodemographic factor associated with UI. Regarding sports factors, a higher weekly training frequency in all disciplines showed a protective effect against UI, especially for SUI and MUI. On the other hand, it was found that a higher weekly running training volume increased the odds of developing MUI. However, despite being statistically significant, the association was nearly null and is unlikely to have clinical relevance.

Recent prevalence studies among power‐and‐weightlifters have reported a prevalence of UI between 32% and 50% [13, 19]. Similarly, this study found that 43.3% of triathletes reported symptoms of UI, suggesting a comparable burden in this sport. Comparing our findings to Yi et al. [15] study, which also evaluated triathletes, we found a a slightly lower prevalence of SUI (37.4% vs. 25.6%). and UUI (16% vs. 3.3%). For MUI the findings of this study revealed a prevalence of 10%, while Yi et al. did not provide data on this condition [15]. Another recent study showed a SUI prevalence of 14.3% among the seven triathletes participating [16]. The rates differences can be explained by the questionnaire used and the age of the participants, respectively. While Yi et al. [15] used the Epidemiology of Prolapse and Incontinence Questionnaire (EPIC), which is more comprehensive, this study was conducted with the ICIQ‐SF questionnaire, a more concise and specific questionnaire for evaluating UI. In the study by Rodríguez‐López et al. [16], the average age of participants was 23.7 ± 7.74 years, while in the present study, the average was 38.1 ± 6.7 years, which may influence the higher prevalence of UI, as increasing age is a known risk factor for UI.

It is well known that increased age, BMI, pregnancy, and parity are associated factors for UI in the general population [2, 4, 20]. The present study evaluated relatively young women with a normal BMI (20 ≤ BMI < 25) [21]. About 39% and 35% of the women had at least one pregnancy and one birth, respectively. Interestingly, the number of pregnancies does not seem to be a predictor of UI, while parity is. Our results demonstrated that parity increased the odds of UI by 57.7% among this population. Corroborating this, Yi et al. also found that SUI was more prevalent in multiparous triathletes (*p* = 0.001) [15]. The physical changes resulting from childbirth, associated with a return to running too early or the absence of adequate post‐partum rehabilitation, can increase the risk of SUI, especially during running [22]. However, in our study, we did not collect information about returning to sport after childbirth.

The most curious finding was that a higher weekly training frequency demonstrated a protective effect against UI and SUI, reducing their likelihood by 24% and 30.5%, respectively, across all triathlon disciplines. Studies have shown that the PFM responds with a pre‐contraction during exercise to counteract increases in IAP [23]. However, two main hypotheses explain why athletes may still experience UI during exercise: (1) high‐impact activities can elevate IAP to levels that exceed the load‐bearing capacity of the pelvic floor, leading to SUI [24], or 2) repetitive increases in IAP during exercise may cause PFM fatigue [25], which could explain why athletes are more likely to experience UI toward the end of a training session [26]. Our findings suggest that increasing the weekly training frequency promotes a training effect for the PFM and prevents exercise‐induced fatigue, as hypothesized by Bø [10].

It is important to note that weekly training frequency should not be analyzed in isolation, but in conjunction with other factors such as training intensity and the type of sport practiced. Literature suggests that high‐impact sports, such as running [27], gymnastics, volleyball, and basketball [27], tend to overload the pelvic floor more than low or moderate‐impact sports like cycling and swimming [27]. In our study, swimming showed the highest weekly frequency and was associated with a protective effect against SUI—Model 1 of the multinomial logistic regression. These results suggest that swimming may play a relevant role in reducing the likelihood of UI. However, it is important to note that, although the statistical association is significant, the certainty of evidence is limited, given the cross‐sectional nature of the study and the absence of direct PFM functional assessment.

The analysis of this study showed that weekly running volume (minutes/week) was a small predictor of MUI (4%). Yi et al. proposed that running is the modality that imposes the most load on the PFM and may contribute to the higher prevalence of pelvic disorders in athletes who perform high running volumes [15]. These results reinforce the complex relationship between the type of exercise, its intensity, and the manifestation of UI. It is important to note that the low number of athletes with MUI (*n* = 9) limits the reliability of statistical associations in multinomial analysis. This may introduce bias and overfitting. Larger sample sizes are needed in future studies to explore UI subtypes more reliably.

In summary, our findings suggest that a higher weekly frequency is associated with a protective effect against UI, possibly because triathletes may achieve a balance between impact (from running) and non‐impact (from cycling and swimming) activities. This balance may explain this protective effect, which contrasts with the increased risk often reported in other athletic populations with high training volumes. The association of running volume with MUI supports the idea that repetitive impact may increase pelvic floor load and dysfunction risk.

For clinicians, these results highlight the potential value of promoting balanced and frequent training routines that incorporate low‐impact activities as part of PFM strategies. These findings also align with the broader evidence that regular physical activity is widely recognized to provide several health benefits. Perhaps suggest an increase in weekly training frequency, rather than volume, could help train the PFM and prevent UI. However, clinicians should remain cautious in interpreting these findings as causal and continue to screen for UI symptoms in triathletes, providing individualized guidance and PFM training where needed. Also, due to the homogeneity of our sample (e.g., age, triathletes), generalizability to broader populations may be limited.

This study has some limitations, including difficulties in collecting data at triathlon events due to the event environment, as well as the lack of interest from athletes in answering the questionnaires during the events. Also, a potential selection bias should be acknowledged, as women experiencing UI or more severe symptoms may have been more inclined to participate in the study. Another limiting factor was the low return rate obtained from online dissemination. The use of questionnaires as a data collection method may introduce bias, as responses are subject to participant subjectivity, recall errors, and interpretation of questions. The variation in the form of responses (online and in‐person) may also have been a limitation; however, this study used a validated and specific questionnaire to assess UI. The cross‐sectional design limits causal inference, and the small number of participants in the UUI (*n* = 3) and MUI (*n* = 9) groups may have limited the statistical power of the adjusted analysis of predictor variables for the outcome type of UI. Therefore, findings related to these subgroups should be interpreted with caution.

## 5 Conclusion

The study revealed that 43.3% of the triathletes experienced UI. Among these, SUI was the most reported type, affecting 25.6% of the participants. Parity was the only sociodemographic factor significantly associated with the development of UI. On the other hand, a higher frequency of weekly frequency in all disciplines appears to reduce the chance of UI occurrence by almost 24%, assuming a protective factor, especially in reducing the chance of SUI by 30.5%. The findings also show that running volume (minutes per week) is associated with a slight increase in the risk of MUI (OR = 1.004), with the effect likely being of little or no clinical relevance.

These findings highlight the importance of considering the specific characteristics of each triathlon discipline in the evaluation and prevention of pelvic disorders. Future investigations should adopt longitudinal or interventional designs with larger, more diverse samples. Incorporating objective assessments of pelvic floor function, individual risk factors (e.g., hormonal status), and biomechanical analyses during sport‐specific activities would provide deeper insights.

## Ethics Statement

The study was approved by the Human Research Ethics Committee of the State University of Santa Catarina (approval no. 6.547.406; CAAE 74004823.0.0000.0118).

## Consent

All participants provided informed consent by signing the TCLE (Termo de Consentimento Livre e Esclarecido)/ICF (Informed Consent Form) at the time of data collection.

## Conflicts of Interest

The authors declare no conflicts of Interest.

## Data Availability

The data that support the findings of this study are available on request from the corresponding author. The data are not publicly available due to privacy or ethical restrictions.
